# Circadian Oscillation Pattern of Endoplasmic Reticulum Quality Control (ERQC) Components in Human Embryonic Kidney HEK293 Cells

**DOI:** 10.5334/jcr.219

**Published:** 2023-04-03

**Authors:** Yalcin Erzurumlu, Deniz Catakli, Hatice Kubra Dogan

**Affiliations:** 1 Department of Biochemistry, Faculty of Pharmacy, Suleyman Demirel University, Isparta, Turkey; 2 Department of Pharmacology, Faculty of Medicine, Suleyman Demirel University, Isparta, Turkey; 3 Department of Bioengineering, Institute of Science, Suleyman Demirel University, Isparta, Turkey

**Keywords:** Circadian rhythm, Endoplasmic reticulum-associated degradation, gp78, Hrd1, Protein-Quality Control

## Abstract

The circadian clock regulates the “push-pull” of the molecular signaling mechanisms that arrange the rhythmic organization of the physiology to maintain cellular homeostasis. In mammals, molecular clock genes tightly arrange cellular rhythmicity. It has been shown that this circadian clock optimizes various biological processes, including the cell cycle and autophagy. Hence, we explored the dynamic crosstalks between the circadian rhythm and endoplasmic reticulum (ER)-quality control (ERQC) mechanisms. ER-associated degradation (ERAD) is one of the most important parts of the ERQC system and is an elaborate surveillance system that eliminates misfolded proteins. It regulates the steady-state levels of several physiologically crucial proteins, such as 3-hydroxy-3-methylglutaryl-CoA reductase (HMGCR) and the metastasis suppressor KAI1/CD82. However, the circadian oscillation of ERQC members and their roles in cellular rhythmicity requires further investigation. In the present study, we provided a thorough investigation of the circadian rhythmicity of the fifteen crucial ERQC members, including gp78, Hrd1, p97/VCP, SVIP, Derlin1, Ufd1, Npl4, EDEM1, OS9, XTP3B, Sel1L, Ufd2, YOD1, VCIP135 and FAM8A1 in HEK293 cells. We found that mRNA and protein accumulation of the ubiquitin conjugation, binding and processing factors, retrotranslocation-dislocation, substrate recognition and targeting components of ERQC exhibit oscillation under the control of the circadian clock. Moreover, we found that Hrd1 and gp78 have a possible regulatory function on Bmal1 turnover. The findings of the current study indicated that the expression level of ERQC components is fine-tuned by the circadian clock and major ERAD E3 ligases, Hrd1 and gp78, may influence the regulation of circadian oscillation by modulation of Bmal1 stability.

**Graphical Abstract:**
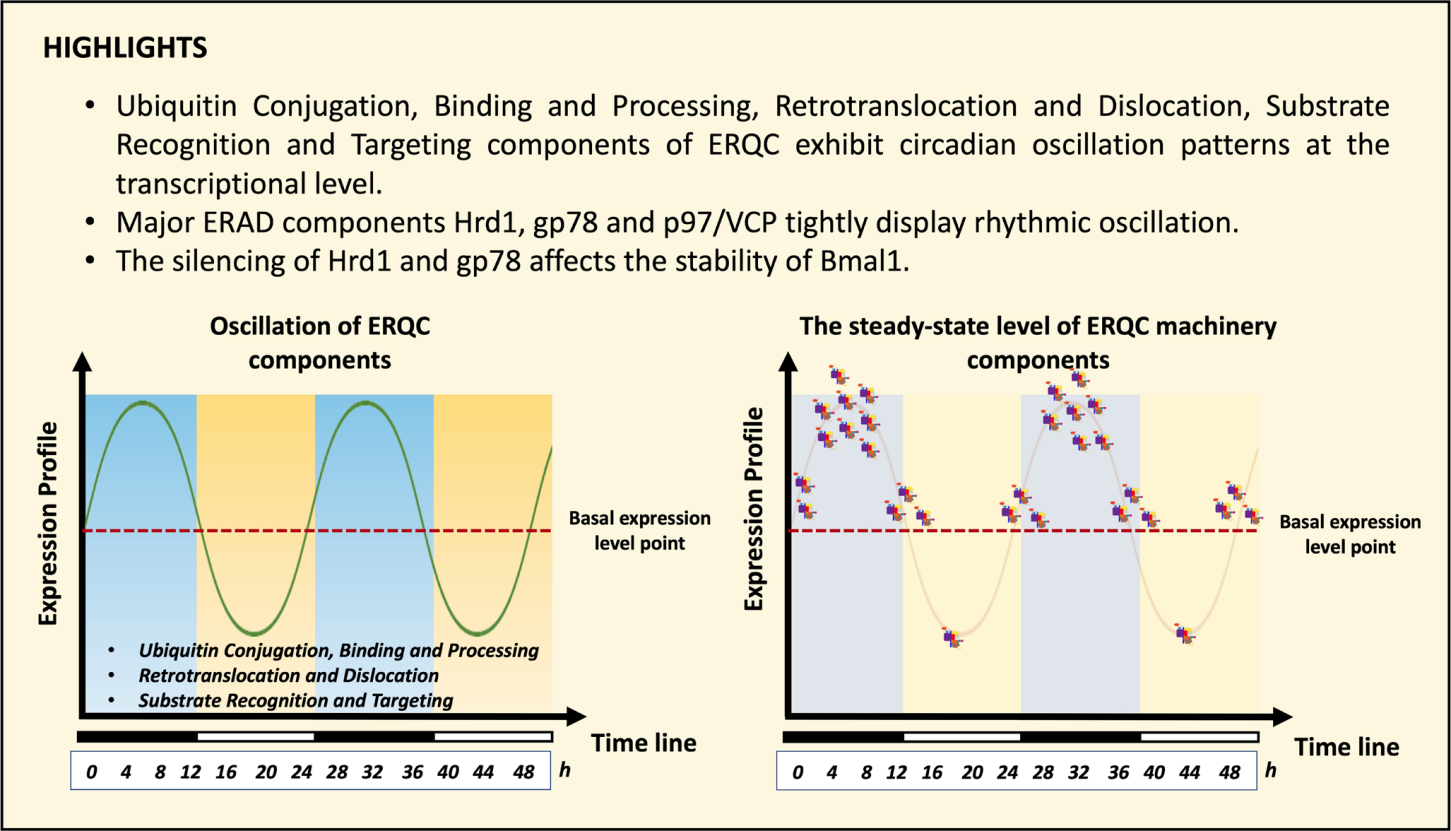
Illustration of oscillation of expression pattern of ERQC components.

## 1. Introduction

All mammalian cells have an endogenous circadian clock that regulates the timing of gene networks at the transcriptional level. Circadian clocks are evolutionarily well-conserved and have been shown to regulate the expression of 43% of all genes [1]. This clock controls the physiological and behavioral processes for a period of close to 24 h in mammals. At the molecular level, the various biological processes, including cell cycle, DNA damage control, autophagy, nutrient metabolism, hormone synthesis and reproductivity are regulated under circadian oscillation [2,3,4]. Circadian rhythm-mediated regulation of the target genes is controlled by the core circadian transcription factors clock circadian regulator (CLOCK)/brain and muscle ARNT-like1 (Bmal1) heterodimer that rhythmically binds to regulatory elements of the target genes. Moreover, negative regulation of the circadian rhythm is controlled with the negative regulators period circadian regulator 1–3 (Per 1–3) and cryptochromes 1 and 2 (CRY 1 and 2), which are regulated with Bmal1/CLOCK-mediated transcriptional program [5]. Furthermore, impairment of cellular circadian rhythmicity has been associated with many diseases, including diabetes, hypertension, depression, obesity, Alzheimer’s disease, cardiovascular diseases, metabolic syndrome and various cancers. Therefore, defining circadian rhythm-related oscillation patterns of molecular mechanisms that have critical roles at the physiological level is crucial. Recent studies have pointed to a crosstalk between the endoplasmic reticulum (ER) related protein quality control mechanism and circadian oscillation [6,7].

ER plays a crucial role in protein synthesis, folding and modification. The protein quality control mechanisms in the ER are well-conserved and sophisticatedly regulated by diverse protein complexes. Newly synthesized proteins undergo a series of post-translational modifications to reach their final form. This process is tightly inspected, otherwise, the accumulation of unfolded, misfolded or improper oligomerized protein forms in the ER leads to an ER stress response. Therefore, cells have evolved an ER quality control (ERQC) system to maintain ER homeostasis. ER-associated degradation (ERAD), one of the most important components of the ERQC system, is the most effective way to remove misfolded proteins [8]. Moreover, it regulates the steady-state levels of physiologically crucial proteins, including monooxygenase cytochrome p450, cholesterol metabolism regulatory proteins 3-hydroxy-3-methylglutaryl-CoA reductase (HMGCR), insulin-induced gene-1 (INSIG1) and apolipoprotein B (ApoB); neurodegenerative disease proteins superoxide dismutase-1 (SOD1) and ataxin-3 and the metastasis suppressor kangai 1(KAI1)/Cluster of differentiation (CD82) as well [9,10,11,12].

ERAD organizationally consists of four functional modules, including substrate recognition, ubiquitination, retrotranslocation and 26S proteasomal degradation and specialized protein groups function in each module such as channel components, chaperones, glycan-specific lectin proteins, glycanase enzymes, retrotranslocation proteins, ubiquitin transferring enzymes; ubiquitin-activating enzyme (E1), ubiquitin-conjugating enzymes (E2) and ubiquitin ligases (E3), ubiquitin chain-elongation enzymes (E4), deubiquitinase enzymes, negative regulator proteins, shuttling complexes and cofactor proteins [13,14].

Currently, dysregulation of ERAD has been associated with almost seventy cellular pathologies such as cardiovascular and neurodegenerative diseases, diabetes, cystic fibrosis and cancer [9,10,11,12,13,15,16,17,18]. Thus, the physiological oscillation of ERQC and the elucidation of its novel regulatory mechanisms are very crucial to understanding the underlying causes of various fatal diseases, including cancer.

Recently, it has been suggested that Hrd1 regulates the circadian clock by coordinating the proteasomal degradation of Bmal1 through ubiquitination [6]. A study of the molecular oscillation regulating the E3 ubiquitin ligase Hrd1-controlled cyclic adenosine monophosphate (cAMP)-responsive element-binding protein H (CREBH)/peroxisome proliferator-activated receptor α (PPARα) transcriptional program in hepatic cells established that Hrd1 and its co-activator Sel1L-controlled CREBH/PPARα transcriptional program regulates hepatic circadian metabolism and function as a major circadian control of lipid mobilization and energy homeostasis [7]. Due to the limited number of studies in the literature, we directed our studies to understand the possible regulation patterns of ERQC under a circadian rhythm. Herein, we have extensively studied the oscillations of numerous ERQC members.

In the present study, we provided a comprehensive approach for the investigation of the circadian rhythmicity of the fifteen crucial ERQC members, including glycoprotein 78 (gp78), hydroxymethyl glutaryl-coenzyme A reductase degradation protein 1 (Hrd1), p97/Valosin containing protein (VCP), small VCP interacting protein (SVIP), degradation in endoplasmic reticulum protein 1 (Derlin1), ubiquitin recognition factor in ER-associated degradation 1 (Ufd1), nuclear protein localization 4 (Npl4), ER degradation enhancing alpha-mannosidase like protein 1 (EDEM1), osteosarcoma amplified 9 (OS9), XTP3B/Endoplasmic reticulum lectin 1 (ERLEC1), suppressor of lin-12-like protein 1 (Sel1L), ubiquitin recognition factor in ER-associated degradation 2 (Ufd2), YOD1 deubiquitinase (YOD1), valosin-containing protein p97/p47 complex–interacting protein, p135 (VCIP135) and family with sequence similarity 8 member A1 (FAM8A1) in human embryonic kidney HEK293 cells. We found ubiquitin conjugation, binding and processing factors, retrotranslocation and dislocation components, substrate recognition and targeting components of ERQC exhibit strong oscillation under the circadian clock. Furthermore, we analyzed the possible regulatory role of gp78 and Hrd1 E3 ligases in controlling the circadian clock through Bmal1 turnover. Our results indicated that the downregulation of the two major E3 ligases of ERAD pathway affected circadian rhythmicity through disruption to Bmal1 levels in human embryonic kidney HEK293 cells. Our data revealed that the ERQC system is tightly regulated under the mammalian circadian clock. The present findings suggest that ERAD may have possible advanced regulatory roles on the circadian clock that need to be analyzed in detail.

## 2. Material and Methods

### 2.1 Materials

All cell culture grade reagents, including media, fetal bovine serum (FBS) and additional growth requirements were obtained from Biological Industries. Horse serum was purchased from Biowest. Rabbit polyclonal antibodies p97/VCP (10736-1-AP)(1:10000), gp78 (16675-1-AP)(1:3000) and Bmal1 (14268-1-AP)(1:2500) were obtained from Proteintech, rabbit monoclonal antibody against Hrd1 (#14773)(1:3000) was provided from Cell Signaling Technology. Mouse monoclonal beta-actin antibody (A5316)(1:10000) was purchased from Sigma Aldrich. Horseradish peroxidase (HRP)-conjugated anti-mouse (#31430)(1:5000) or anti-rabbit (#31460)(1:5000) IgG (H+L) was purchased from Thermo Scientific. Negative control dicer-substrate small interfering RNA (DsiRNA) DsiRNA (#51-01-14-04) and custom design DsiRNAs against Hrd1 (#23-11-61-519) and gp78 (#22-97-42-985) were obtained from Integrated DNA Technologies.

### 2.2 Cell culture

Human embryonic kidney cell line HEK293 (CRL-1573™) was obtained from American Type Culture Collection (ATCC). Cells were propagated and routinely passaged in Dulbecco’s Modified Eagle Medium (DMEM) containing 10% FBS, 5 mg ml^–1^ penicillin/streptomycin and 2 mM L-glutamine (Biological Industries). Cells were kept in a humidified atmosphere of 5% CO_2_ and 95% air at a constant temperature of 37°C. The absence of mycoplasma contamination was routinely confirmed by using EZ-PCR™ Mycoplasma Detection Kit (Biological Industries).

### 2.3 Transfection

Negative control DsiRNA (#51-01-14-04), Hrd1 DsiRNA (hs.Ri.SYVN1.13.1) and gp78 DsiRNA (hs.Ri.AMFR.13.1) were ordered from Integrated DNA Technologies (IDT). Transfection application was performed with Xfect™ RNA transfection (#631450) (Takara) reagents according to the manufacturer’s instructions. Cells were transfected with 5nM negative control DsiRNA and 5nM Hrd1 or 5nM gp78 DsiRNA for 36 h before being subjected to serum shock for 2 h for circadian synchronization protocol. Negative control DsiRNA was used as a negative control application. Cells were grown in 60 mm cell culture dishes and transfection was started when the cells were 80–90% confluent. DsiRNA and transfection polymer were separately homogenized with reaction buffer in tubes and then tubes were fused and incubated for 10 min at room temperature to allow nanoparticle complexes to form. The transfection solution was gently dropwise to the cell culture medium.

### 2.4 *In vitro* circadian synchronization of HEK293 cells

Cells were seeded on 10mm cell culture dishes. At confluence, the cells were propagated for 6 days and then cells were subjected to 50% horse serum (serum shock) or 10% FBS containing regular media for 2 h for circadian synchronization [19]. After serum shock synchronization, the shock medium was replaced with a serum-free medium. Cells were collected at 4 h intervals for 48 h and lysed for RNA and/or protein isolation. HEK293 cells were transfected with negative control DsiRNA (5nM) and Hrd1 (5nM) or gp78 (5nM) DsiRNA for 36 h before being subjected to serum shock for 2 h for circadian synchronization protocol.

### 2.5 RNA extraction and cDNA synthesis

Total RNA was extracted from cell pellets by using Aurum™ Total RNA Mini Kit (Bio-Rad) according to the manufacturer’s instructions. RNA concentration and purity were determined by a micro-spectrophotometer (Allsheng). Total RNA (1 μg) was reverse transcribed with iScript cDNA Synthesis kit by using oligo dT and random primers (Bio-Rad). The reaction mix was incubated for 5 min at 25°C, for 20 min at 46°C and 1 min at 95°C.

### 2.6 Reverse Transcription PCR (RT-PCR) and agarose electrophoresis

Primers were designed specifically for genes of interest, including Hrd1, gp78, p97/VCP, SVIP, Ufd1, Npl4, EDEM1, SEL1L, OS9, OTUB1, VCIP135, Ufd2, XTP3B, FAM8A1, Derlin1, YOD1, PER1 and TBP. All primer sequences are presented in the supplementary data section. cDNA samples were analyzed in triplicates for each PCR. The expression of TATA-Box Binding Protein (TBP) was used for normalization. Specific primers were used for the PCR amplification with Taq DNA polymerase (Thermo Scientific). The following PCR conditions were used: pre-denaturation at 95°C for 5 min, followed by 40 cycles of denaturation 30 s at 95°C, annealing 30 s at 60°C and extension 30 s 72°C, final extension 2 min 72°C. Thereafter, we separated the PCR products by electrophoresis on 2% agarose gel containing ethidium bromide (Et-Br) and visualized by UV-transilluminator unit of iBright CL1000 (Thermo Scientific).

### 2.7 Protein Preparation and Western blot analysis

Cells were lysed in Radioimmunoprecipitation assay (RIPA) buffer (1xPBS, 1% nonidet P-40, 0.5% 7-DOC and 0.1% SDS, pH 8.0). After the removal of the insoluble phase by centrifugation at 14.000 rpm for 20 min at 4°C, total protein concentrations were determined by the bicinchoninic acid assay (BCA) (Thermo Scientific). Typically, 25–40 μg of total cellular protein was used in immunoblotting studies. Samples were denatured in 4× Laemmli buffer at 70°C for 15 min and were separated on hand-cast polyacrylamide gels. Gels were transferred to an Immobilon®-P polyvinylidene fluoride (PVDF) membrane (Bio-Rad). The membrane was blocked in 5% skim milk in phosphate-buffered saline (PBS) containing 0.1% Tween (PBS-Tween) for 1 h at room temperature and then incubated with the primary antibody prepared in PBS–Tween containing 5% skim milk for 1–2 h at room temperature or at 4°C overnight. Secondary antibodies were applied for 1 h at room temperature. Protein bands were visualized using Clarity™ western enhanced chemiluminescence (ECL) solution (Bio-Rad) by iBright CL1000 (Thermo Scientific). Densitometric analysis of protein bands was carried out by using Image Studio™ Lite (LI-COR®).

### 2.8 Curve fitting analysis

The circadian oscillation pattern of ERAD genes was conducted using a non-linear curve fitting algorithm using GraphPad Prism and MATLAB software, which fitted a sinusoid function [A*sin(Bt + c)] to the data, including the three replicates. Genes showing an *R^2^* correlation greater than 0.8 in non-linear curve fitting analyzes were kept.

### 2.9 Statistical Analysis

Results were presented as means ± standard deviation (SD). The statistical significance of differences between groups was determined by a two-tailed equal variance Student’s t-test with a confidence interval, minimum, of 95% using GraphPad Prism 5 software. Values of p < 0.05 were considered significant.

### 2.10 Ethical Approval

This study does not require any ethical permission.

## 3. Results

### 3.1 Ubiquitin conjugation, binding and processing components of ERQC expression exhibit circadian rhythmicity

First, we examined the 48 h rhythmic expression profile of ubiquitin conjugation, binding and processing components of ERQC. These included Hrd1, gp78, VCIP135, YOD1, Ufd2 and FAM8A1 in human embryonic kidney HEK293 cells. To evaluate the relationship between ERQC components and circadian rhythmicity, we programmed the HEK293 cells according to the previously described protocol [19].

**Figure 1:**
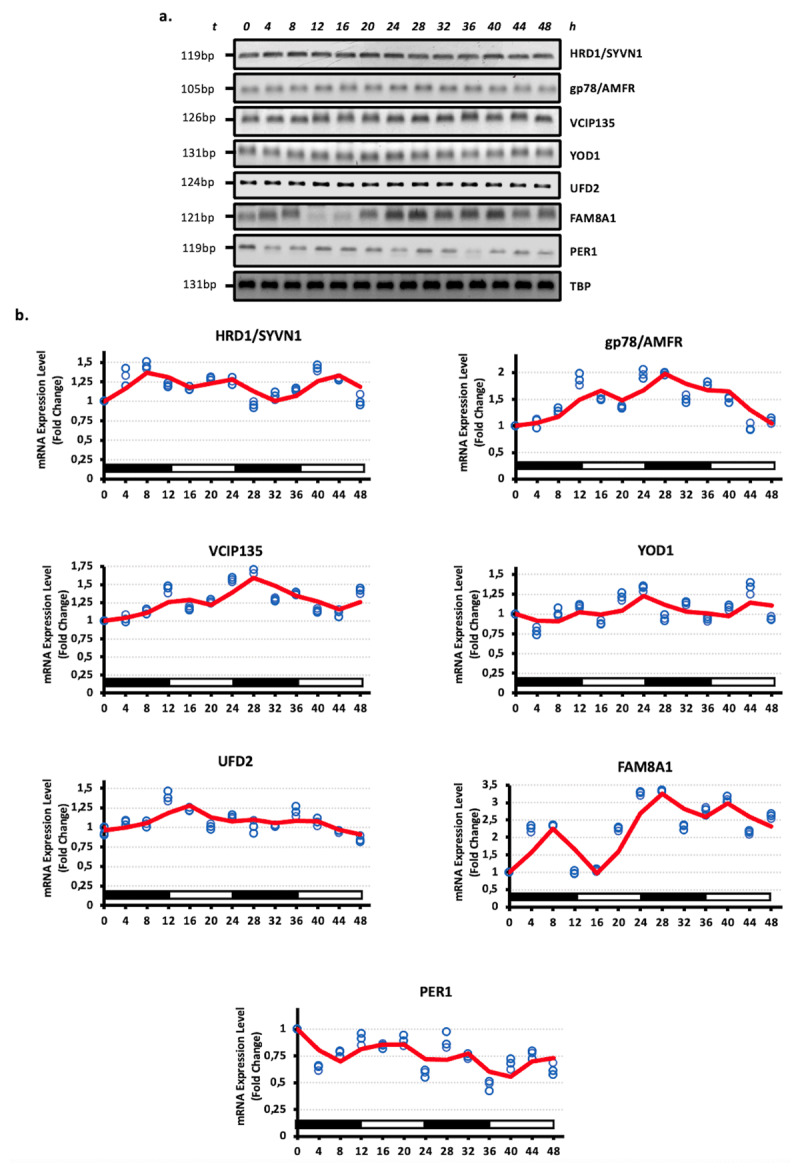
**Ubiquitin conjugation, binding and processing components of ERQC exhibit circadian rhythmicity. a.** Rhythmic expression levels of Hrd1, gp78, VCIP135, YOD1, Ufd2, FAM8A1 and PER1 genes in the HEK293 cells across a 48 h circadian cycle. The total RNAs were isolated from the HEK293 cells collected every 4 h during a 48 h circadian period and reverse transcribed. The amplified PCR products were visualized on agarose gels. b. Relative gene expression quantities corresponding to three biological replicates were collected every 4 h (blue circles). The intensity of the bands was analyzed densitometrically and TBP was used as an internal control. Fold changes in mRNA expression levels were determined by comparison to the expression level at 0 h. Oscillation (represented as a continuous curve) was modeled, via curve fitting analysis. A nonlinear curve fitting analysis was conducted, which fitted a sinusoid function [A*sin(Bt + c)] to the data including the replicates. Genes showing an *R^2^* correlation greater than 0.8 in non-linear curve fitting analyzes were kept. (n = 3).

For this purpose, serum shock was applied to the HEK293 cells. The success of serum synchronization was confirmed by examining the oscillation of period circadian regulator 1 (PER1) expression. As expected PER1 exhibited rhythmic oscillation (Figure 1a, b). All tested ERQC components, including HRD1/SYVN1, gp78/AMFR, VCIP135, YOD1, UFD2 and FAM8A1 exhibited a typical circadian rhythmic pattern, which peaked at 8–12 h, 24–28 and 40–44 h and troughed at 16–20 and 32–36 h (Figure 1a, b). Our results for all the members we tested showed that the ubiquitin conjugation, binding and processing components of the ERQC exhibited a synchronous expression pattern with expression profiles increasing and decreasing with rhythm. These results indicated that members of the ubiquitin conjugation, binding and processing components of ERQC tightly exhibited oscillation profiles under the circadian clock.

### 3.2 Member of the retrotranslocation and dislocation partners of ERQC to display oscillation under the circadian clock

**Figure 2:**
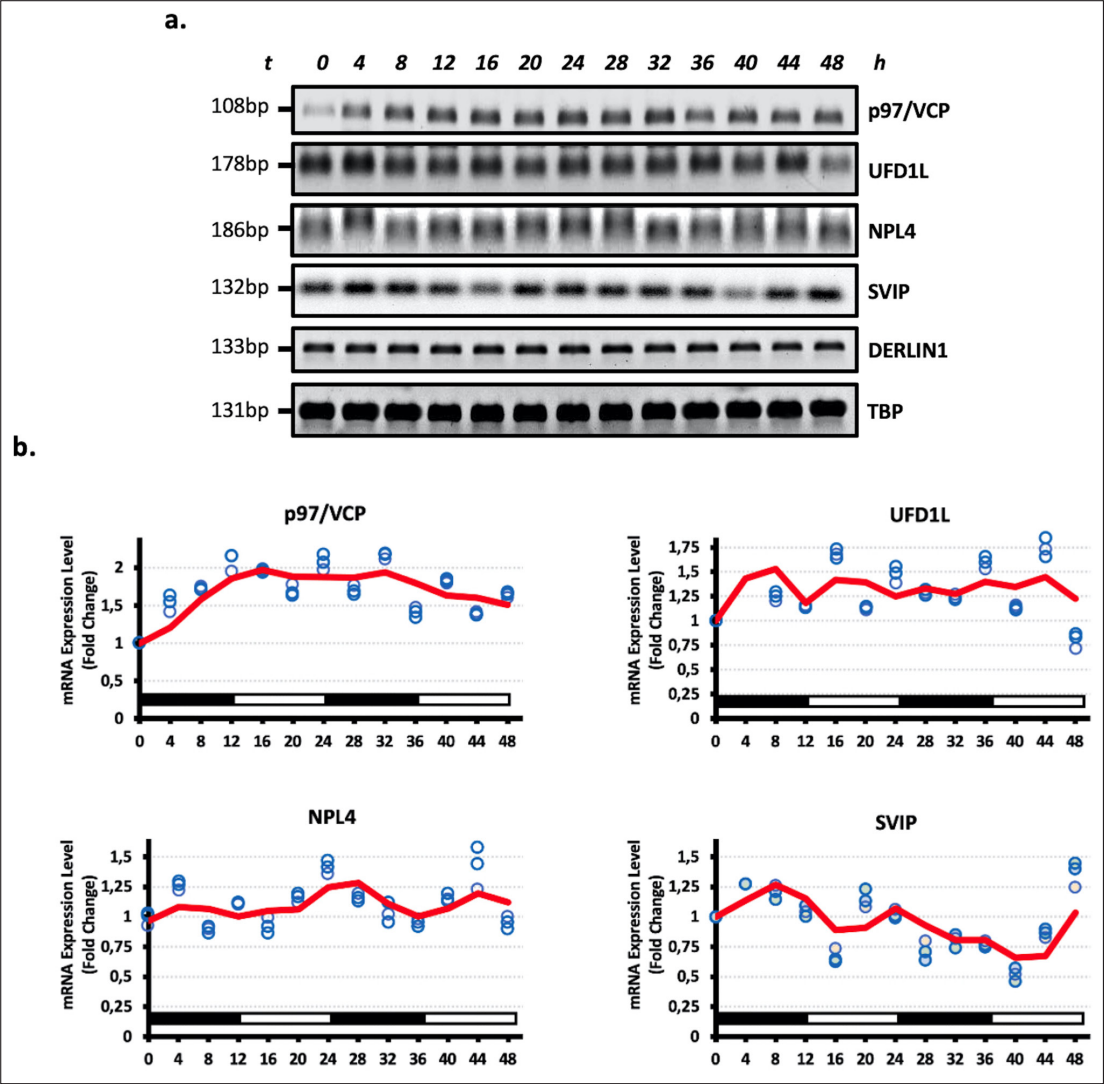
**The members of the retrotranslocation and dislocation components of ERQC exhibit a rhythmical expression pattern. a.** mRNA expression levels of p97/VCP, Ufd1, Npl4, SVIP and Derlin1 in the HEK293 cells across a 48 h circadian cycle. The total RNAs were isolated from the HEK293 cells collected every 4 h during a 48 h circadian period and cDNAs were synthesized. Genes of interest were amplified by PCR and visualized on agarose gels. b. Relative gene expression quantities corresponding to three biological replicates were collected every 4 h (blue circles). The intensity of the bands was analyzed densitometrically and normalized to TBP expression. Fold changes in mRNA expression levels were determined by comparison to the expression level at 0 h. Oscillation (represented as a continuous curve) was modeled, via curve fitting analysis. A nonlinear curve fitting analysis was conducted, which fitted a sinusoid function [A*sin(Bt + c)] to the data including the replicates. Genes showing an *R^2^* correlation greater than 0.8 in non-linear curve fitting analyzes were kept. (n = 3).

The retrotranslocation and dislocation step of ERAD is crucial for delivering substrate proteins to the 26S proteasome. The member of the large ATPase associated with diverse cellular activities (AAA+) family, p97/VCP and its accessory factors, responsible for recognition of substrates, Ufd1-Npl4 modulate the retrotranslocation [20,21]. Besides, SVIP as a VCP-interacting protein is an endogenous negative regulator of ERAD [22]. To understand the possible oscillation pattern of retrotranslocation and dislocation partners of ERQC, we examined circadian rhythmic expression profiles of the genes p97/VCP, Ufd1, Npl4 and SVIP in HEK293 cells. Similarly, mRNA expression levels of all tested ERQC components displayed circadian rhythmicity with a peak at 8–12 h, 24–28 h and 40–44 h and troughed at 16–20 h and 32–36 h (Figure 2a, b). However, Derlin1, which is a functional lumenal subunit of ERAD, not displayed circadian rhythmicity in human embryonic kidney HEK293 cells under the circadian clock (Figure 2). Moreover, it was remarkable that the expression levels of SVIP, the first identified endogenous inhibitor of ERAD, showed a parallel pattern to other ERAD components, including Hrd1, gp78 and p97/VCP.

### 3.3 Critical elements of ERQC for substrate recognition and targeting show oscillation patterns

**Figure 3:**
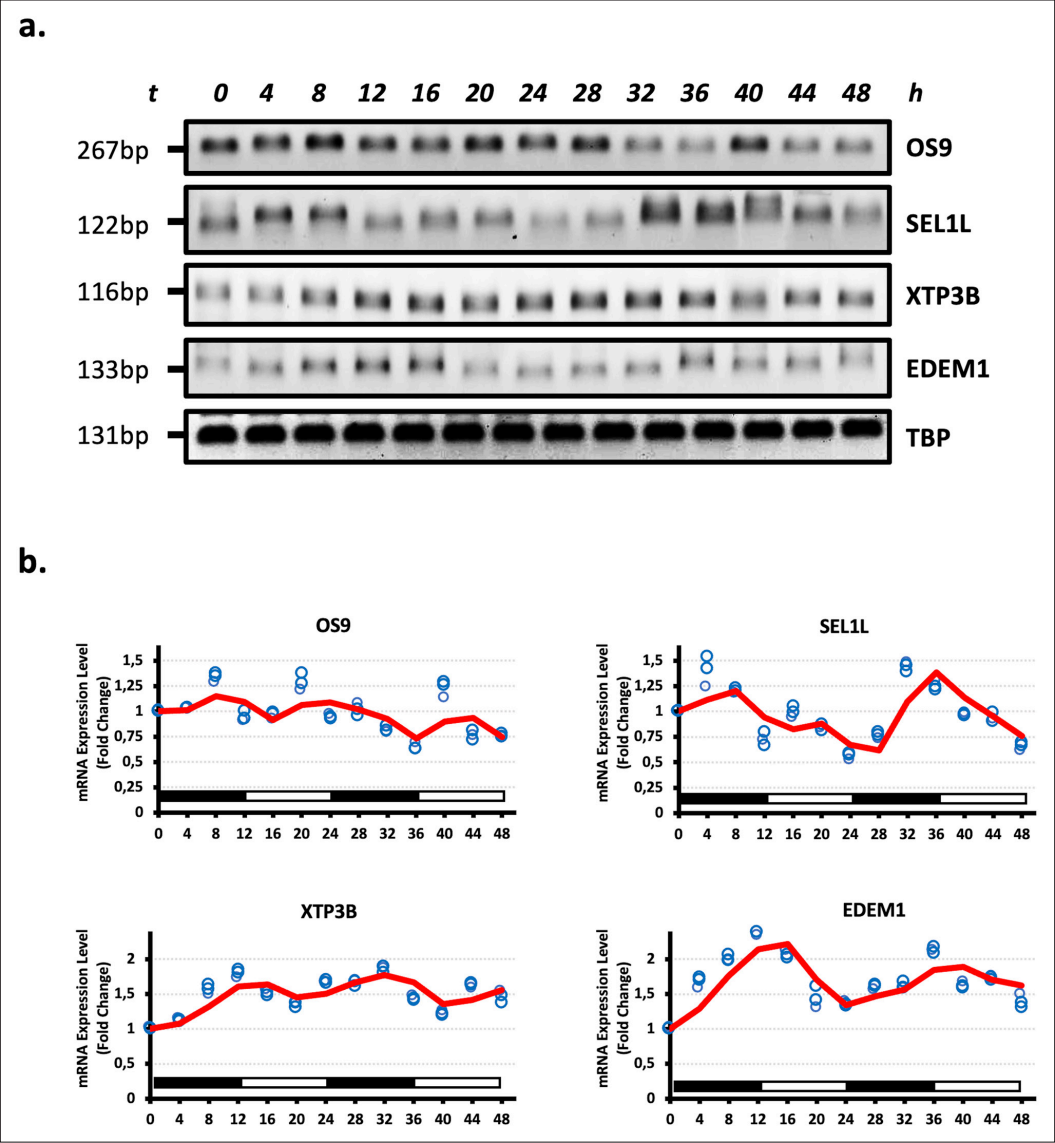
**Rhythmic oscillation of substrate recognition and targeting components of ERQC. a.** Rhythmic expression levels of OS9, SEL1L, XTP3B and EDEM1 genes in the HEK293 cells across a 48 h circadian cycle. The total RNA was extracted from the HEK293 cells collected every 4 h during a 48 h circadian period and reverse transcribed. RT-PCR was performed to measure gene expression. TBP served as an internal control. The amplified PCR products were visualized on agarose gels. b. Relative gene expression quantities corresponding to three biological replicates were collected every 4 h (blue circles). Densitometrically analyzed bands were normalized to TBP expression. Fold changes in mRNA expression levels were determined by comparison to the expression level at 0 h. The relative expression level of each gene is plotted in the graph. Oscillation (represented as a continuous curve) was modeled, via curve fitting analysis. A nonlinear curve fitting analysis was conducted, which fitted a sinusoid function [A*sin(Bt + c)] to the data including the replicates. Genes showing an *R^2^* correlation greater than 0.8 in non-linear curve fitting analyzes were kept. (n = 3).

To investigate the oscillation rhythm of the substrate recognition and targeting factors of ERQC, including OS9, Sel1L, XTP3B and EDEM1; HEK293 cells were programmed to circadian rhythmicity by serum shock. OS9 and XTP3B function as ER lectins, which are recognized specific glycan chains on the substrate molecule and coordinate the channel complex [23]. Sel1L is a critical adaptor protein for Hrd1-mediated ERAD that stabilized the Hrd1 in the ER membrane [24,25]. Furthermore, EDEM1, which functions as an ER mannosidase, extract the misfolded proteins from the repetitive folding cycle and targets them for proteasomal degradation by ERAD [26,27]. Consistent with the other all tested ERQC components, it was revealed that substrate recognition and targeting components of ERQC, including OS9, Sel1L, XTP3B and EDEM1 exhibit rhythmic oscillation under the circadian clock for 48 h. All members of the ERQC components acted in tune with the circadian fluctuation with a peak at 8–12 h, 24–28 h and 40–44 h and troughed at 16–20 h and 32–36 h (Figure 3).

### 3.4 Hrd1, gp78 and p97/VCP proteins exhibit rhythmic oscillation

**Figure 4:**
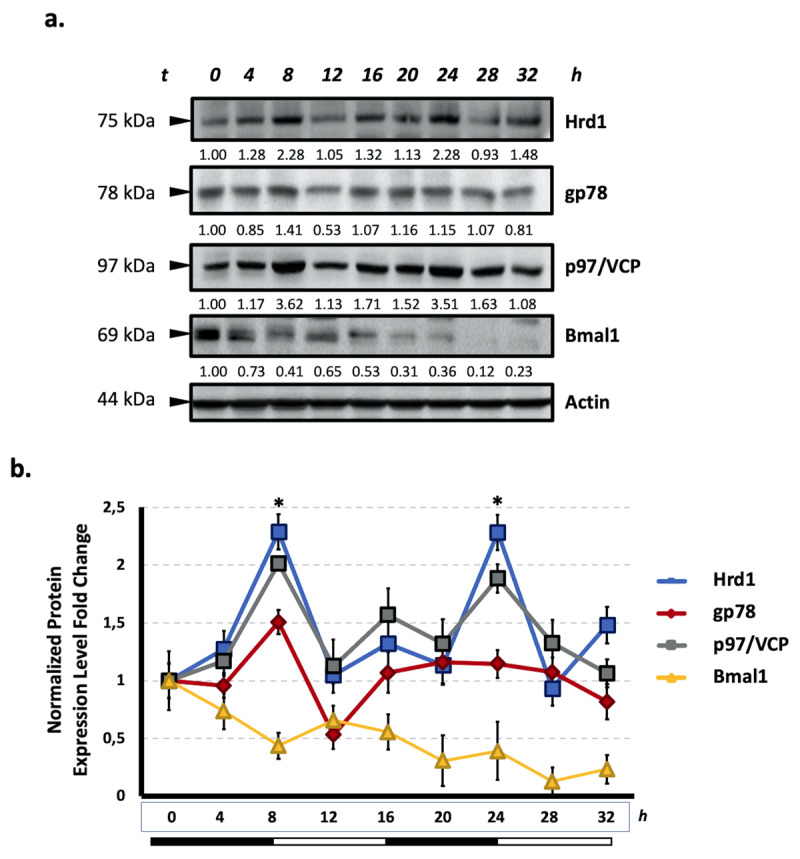
**Hrd1, gp78 and p97/VCP expression exhibit circadian rhythmicity in HEK293 cells. a.** The rhythmic protein accumulation levels of Hrd1, gp78, p97/VCP and Bmal1 in HEK293 cells. Samples were collected every 4 h in a 32 h circadian period. Beta-actin was used as a loading control. b. The intensity of the protein bands was densitometrically analyzed and data represent means ±SEM in the graph (n = 3) * p < 0.05.

After determining the circadian oscillations of the ERQC components at the mRNA level, we examined the circadian rhythmicity of Hrd1, gp78 and p97/VCP at the protein level. Our results showed that protein levels of Hrd1, gp78 and p97/VCP exhibited a continual oscillation pattern at 12 h periods (Figure 4a). In addition, it was observed that Bmal1 levels were down at the peak points of all tested ERQC members (Figure 4a, b).

### 3.5 Two major E3 ligases for ERAD control the circadian clock by regulating the stabilization of Bmal1

**Figure 5:**
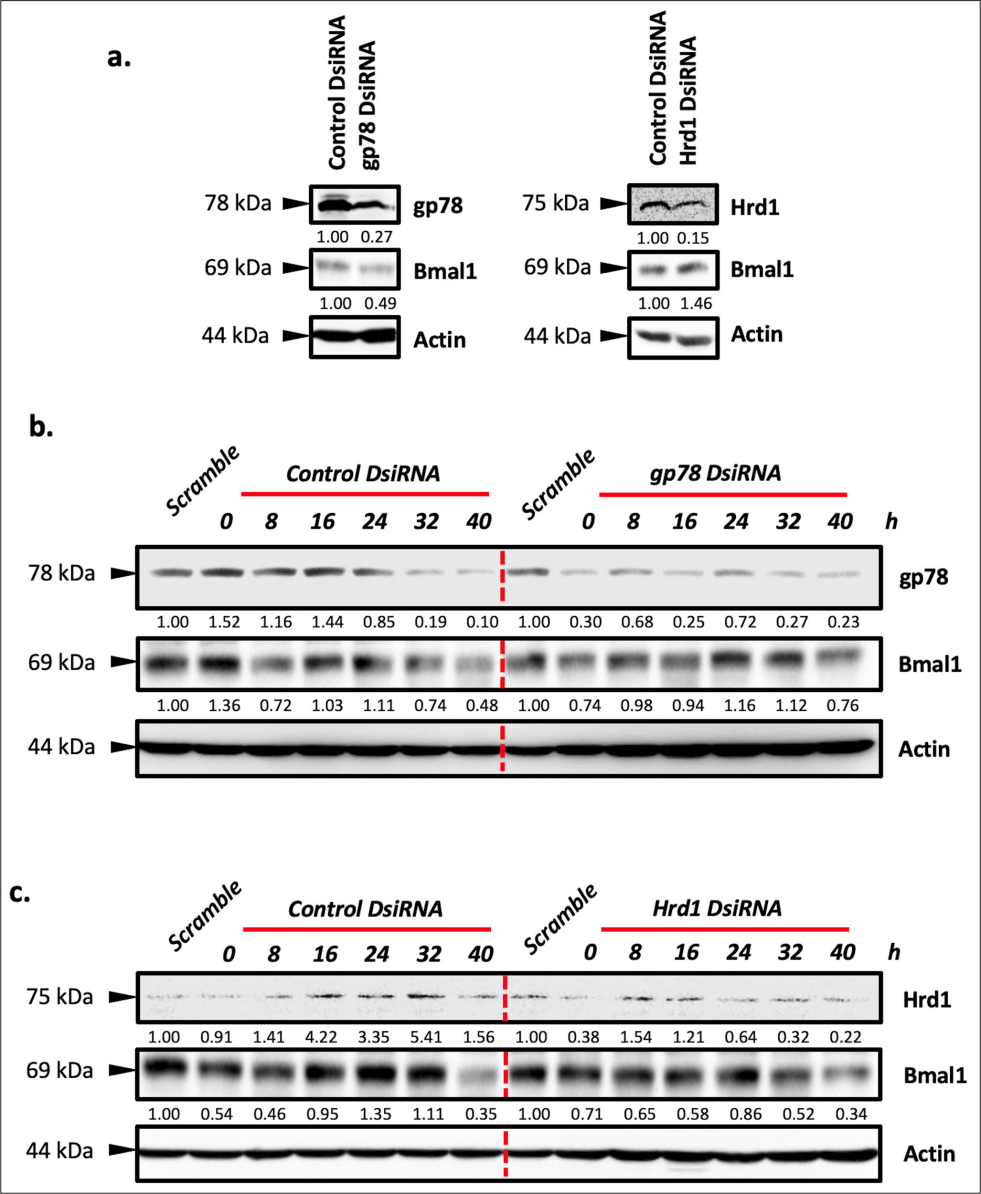
**Knockdown of Hrd1 and gp78 disrupt the circadian oscillation of Bmal1. a.** HEK293 cells were transfected with DsiRNA for the indicated genes. The silencing efficiency of Hrd1 and gp78 proteins and their effects on Bmal1 levels were analyzed by immunoblotting. b, c. Hrd1 or gp78 were silenced by 5nM DsiRNA for 36 h and then synchronization protocol was followed as explained in the Material and Methods section. Samples were collected every 8 h in a 40 h circadian period. Beta-actin was used as a loading control. The scramble group was used as a non-synchronized group. The blots were densitometrically quantified and normalized by using beta-actin expression level.

Next, we examined whether Hrd1 and gp78, well-studied major ERAD E3 ligase enzymes, can regulate the expression of Bmal1 and affect circadian rhythmicity in human embryonic kidney HEK293 cells under the circadian clock. To evaluate the effect of Hrd1 and gp78 on driving the expression level of Bmal1, we knockdown the Hrd1 and gp78 by using DsiRNA in HEK293 cells. Immunoblotting assay results indicated that basal expression levels of Hrd1 and gp78 decreased by more than 50% compared to the control group (Figure 5a). Knockdown of the Hrd1 and gp78 significantly affected the steady-state level of Bmal1 (Figure 5b, c). Moreover, the knockdown of the Hrd1 and gp78 disrupted the oscillation pattern of Bmal1 expression under the circadian clock (Figure 5b, c). Together our results suggest that Hrd1 and gp78 may regulate circadian oscillation by controlling the proteasomal degradation of Bmal1.

## 4. Discussion

The circadian clock regulates the “push-pull” of the molecular signaling mechanisms that arrange the rhythmic organization of the physiology to maintain cellular homeostasis [27]. In mammals, molecular clock genes, Bmal1 and Clock, which function as transcriptional activators, rhythmically regulate the expression of their repressor proteins, the period (PER) and cryptochrome (CRY) [28]. The circadian clock is evolutionary conserved molecular machinery and optimizes various biological processes, including the cell cycle, DNA damage control, autophagy, nutrient metabolism, hormone synthesis and reproductivity as well [29]. Strikingly, disruption of circadian rhythmicity in the cells increases susceptibility to multiple diseases, including diabetes, hypertension, depression, Alzheimer’s disease, cardiovascular diseases, metabolic syndrome and cancer as well [30]. Especially, functioning dysregulation of the circadian clock due to changes or mutations in the expression patterns of clock genes has been associated with the development of tumorigenesis [31]. Therefore, it is critical to mechanistically explain the dynamic crosstalk between the circadian rhythm and the physiologically pivotal molecular mechanisms, particularly the ERQC system.

The ER is the main portal of entry into the secretory pathway. In eukaryotes, more than one-third of the total proteins and all transmembrane proteins are synthesized in the ER and targeted to the secretory pathway [32]. For this purpose, newly synthesized nascent polypeptide chains are promptly undergoing folding and post-translational modification processes following their entry into the ER to achieve their biologically suitable forms. Only reached the correct conformational maturity proteins are allowed to exit the ER. Therefore, an ERQC system in cells has been developed to guarantee high fidelity of protein synthesis and maturation. ERQC continuously controls the folding state of proteins and discriminates between misfolded, unfolded or improperly oligomerized protein forms. It only ensures the transportation of properly maturated proteins to their final destination, thereby preserving ER homeostasis. Proteins that cannot reach their final form are degraded by a mechanism called ERAD to protect the cell from proteotoxicity [8]. ERAD specifically recognizes the unfolded, misfolded or unassembled polypeptide chains and translocates them to the cytosol for degradation through the 26S proteasome [13,33]. Moreover, ERAD is an important physiological regulator for cells to orchestrate the steady-state levels of normal protein, including monooxygenase cytochrome p450, cholesterol metabolism regulatory protein HMGCR, INSIG-1 and ApoB; neurodegenerative disease proteins SOD1 and ataxin-3; and the metastasis suppressor KAI1/CD82, phosphatase and tensin homolog (PTEN) and the circadian clock associated Bmal1 [6,9,10,11,12,34,35].

ERAD is a complicated and highly regulated mechanism involving multiple modules, classified as substrate recognition, ubiquitination, retrotranslocation and 26S proteasomal degradation [13]. Numerous protein components are involved in each module. In ERAD, many key protein groups such as channel components, chaperones, substrate binding and recruitment factor Sel1L; glycan binding lectin, OS9 and XTP3B1; glycanase enzyme EDEM1, retrotranslocation complex members, Derlin1, p97/VCP, Ufd1 and Npl4; ubiquitin transferring enzymes, ubiquitin-activating enzyme, ubiquitin-conjugating enzymes and ubiquitin ligases, gp78 and Hrd1; ubiquitin chain-elongation enzyme Ufd2, deubiquitinase enzymes, YOD1 and VCIP135; endogenous negative regulator protein of ERAD SVIP; shuttling complexes and cofactor protein FAM8A1; accompanying the process are working synchronously [13,33]. A growing number of studies have revealed that several pathologies are associated with the dysregulation of ERQC, including diabetes, cardiovascular diseases, neurodegenerative diseases and many types of cancers [17,18,36,37].

Hrd1 is an ER transmembrane E3 ubiquitin ligase, which regulates the degradation of numerous substrates, some of which are associated with critical regulators and disease-related proteins. Furthermore, it is essential for the ERAD of gamma-aminobutyric acid (GABA) B (GABAB) receptor, nuclear factor erythroid 2-related factor 2 (Nrf2), Parkin-associated endothelial receptor-like receptor (Pael-R), mutant tyrosinase and z-variant antitrypsin (AAT). Additionally, it has been reported that Hrd1 is responsible for the regulation of steady-state levels of ERN1 (ER to Nucleus Signaling-1), NFE2L1 (Nuclear Factor, Erythroid 2 Like 1), MAPT (microtubule-associated protein tau), cytosolic p53 and polyglutamine-extended Huntington protein (polyQ-Htt) [38,39,40,41,42,43,44,45,46]. Considering to critical roles of ERQC on cellular physiology, it is important to characterize its connection with the circadian rhythm and to show its possible regulatory roles on this molecular clock.

Recent studies draw attention to the regulatory relationships between some members of the ERQC system and the circadian rhythm. Hrd1-mediated regulation of the circadian clock through ubiquitination of Bmal1 has been addressed in several recent studies [6,7,47]; however, less is known about the oscillation of ERQC components and their possible regulatory roles in circadian rhythmicity. A study of molecular oscillation regulating the E3 ubiquitin ligase Hrd1-controlled CREBH/PPARα transcriptional program in hepatic cells established that Hrd1 and its co-activator Sel1L-controlled CREBH/PPARα transcriptional program regulates hepatic circadian metabolism and function as a major circadian control of lipid mobilization and energy homeostasis [7]. Moreover, knockdown and overexpression of Hrd1 revealed that it regulates the stability of Bmal1 and also it controls the amplitude of circadian oscillations in mammalian cells [6]. Due to this limited number of studies in the literature, we directed our studies to understand the possible regulation patterns of ERQC under a circadian rhythm and we have extensively studied the oscillations of numerous ERQC members.

Our results indicated that the mRNA expression level of ubiquitin conjugation, binding and processing members of ERQC, including gp78 and Hrd1, VCIP135, YOD1, Ufd2 and FAM8A1, displayed oscillation patterns in serum shock-mediated circadian rhythmically programmed HEK293 cells (Figure 1a, b). Furthermore, other crucial components of ERQC, p97/VCP, Ufd1, Npl4 and SVIP, exhibited rhythmic profiles, whereas Derlin1 did not appear in an oscillation pattern (Figure 2a, b). Similarly, substrate recognition and targeting elements, including OS9, Sel1L, XTP3B and EDEM1, exhibited oscillation patterns in HEK293 cells (Figure 3a, b).

Next, we investigated the protein expression level of Hrd1, gp78 and p97/VCP under a circadian rhythm. Consistent with the mRNA expression data, immunoblotting results showed that Hrd1, gp78 and p97/VCP exhibited continual oscillation patterns at 12 h periods under circadian rhythm in HEK293 cells (Figure 4a, b). In addition, it was observed that Bmal1 levels decreased at the point where Hrd1, gp78 and p97/VCP protein levels peaked. These results suggest a tight relationship between ERAD components and Bmal1 regulation.

Lastly, we tested the effect of the knockdown of gp78 and Hrd1 on circadian oscillation, our data showed that silencing of Hrd1 and gp78 expression disrupts the circadian oscillation in HEK293 cells (Figure 5b, c). Additionally, we found that DsiRNA mediated-downregulation of gp78 decreased Bmal1 levels, whereas knockdown of Hrd1 significantly increased the steady-state level of Bmal1 in HEK293 cells (Figure 5a). Very recently, Guo et al. showed that Hrd1 controls the stabilization of Bmal1 by ubiquitination; our Hrd1-knockdown results confirmed these data [6]. Additionally, our gp78-knockdown findings suggest that gp78 may affect steady-state levels of Bmal1. However, further analyzes are needed to confirm this very limited experimental result.

In summary, the present results suggest that ubiquitin conjugation, binding and processing factors, retrotranslocation and dislocation components, substrate recognition and targeting proteins of ERQC exhibited oscillation under a circadian rhythm. Besides, the major E3 ligase enzymes of ERAD, gp78 and Hrd1 directly or indirectly may regulate the circadian clock in HEK293 cells by regulating the Bmal1 levels.

Since 2003, targeting the ubiquitin-proteasome system, several proteasome inhibitors, bortezomib, carfilzomib and ixazomib, have been approved by Food and Drug Administration (FDA) for the treatment of multiple myeloma [48]. Today, the combined applications of developed new proteasome inhibitors with conventional drugs are thought to be promising for the treatment of many pathologies, including cancer. As a conclude, mechanistically understanding and characterization of circadian clock-mediated regulation of the master regulatory mechanisms of mammalian physiology, including ERQC, will play an essential role in both optimizing the timing of drug administration and presenting new drug target opportunities.

## Additional File

The additional file for this article can be found as follows:

10.5334/jcr.219.s1 Information of Primer Sequences. Supplementary Data Primer Sequences.
