# Supplementary material for: Circadian Oscillation Pattern of Endoplasmic Reticulum Quality Control (ERQC) Components in Human Embryonic Kidney HEK293 Cells
Source: J Circadian Rhythms. 2023 Apr 3;21:1. doi: 10.5334/jcr.219 (PMC10077977; doi:10.5334/jcr.219)
Supplement: Information of Primer Sequences. — Supplementary Data Primer Sequences. [file jcr-21-219-s1.pdf]

Gene Name (NCBI Gene ID)	Primer Sequence	
**HRD1/SYVN1 (ID:84447)**	***Forward***	5’-CTCACGCCTACTACCTCAAAC-3’
	Reverse	5’-CTTGCCCAGAAGGAAGACAA-3’
**GP78/AMFR (ID:267)**	***Forward***	5’-TTCGATGGGTCTCGGATTGC-3’
	Reverse	5’-ATTGAGCTGGGAGTTGCTGG-3’
**P97/VCP (ID:7415)**	***Forward***	5’-GTGGTTTGGGGAGTCTGAGG-3’
	Reverse	5’-CGAGCCTTGGCAATCGAATC-3’
**UFD1L (ID:7353)**	***Forward***	5’-ACACAGTACCGCTGCTTCTC-3’
	Reverse	5’-ATCAGCCACAAACTCCAGCA-3’
**NPL4 (ID:55666)**	***Forward***	5’-TGAAGCGGATCACAGCAACA-3’
	Reverse	5’-GGAACAACAAATCGCCATGCT-3’
**UFD2 (ID:9354)**	***Forward***	5’-CATGCTCACCACCTCTCTATTC-3’
	Reverse	5’-GGGAATCTTTCCATTGGCTTTC-3’
**OS9 (ID:10956)**	***Forward***	5’-CAGAGGCAAGCCGTAGACTC-3’
	Reverse	5’-GGGAGCACCAGAAGCTGAAT-3’
**FAM8A1 (ID:51439)**	***Forward***	5’-CCCTGATTTCCACACACTAAGA-3’
	Reverse	5’-CAGAGTAGTCACCTGCATCAC-3’
**YOD1 (ID:55432)**	***Forward***	5’-GAGCTACTAACCATCAGCTCTTC-3’
	Reverse	5’-GCACAGACCATGCTCCTAAT-3’
**VCIP135 (ID:80124)**	***Forward***	5’-CGATACGGGTTTCCTCCTAAAG-3’
	Reverse	5’-CTGACCACCTTCAGCTTTACT-3’
**SVIP (ID:258010)**	***Forward***	5’-CAAAAAGAGGCTGCATCTCGG-3’
	Reverse	5’-AACTGTCCACCTAAGTCCACC-3’
**SEL1L (ID:6400)**	***Forward***	5’-CAGATGACCTTGACTGACCTAAA-3’
	Reverse	5’-TGCCAGAAAGAGGCTAAGTG-3’
**XTP3B (ID:27248)**	***Forward***	5’-GGTGTCGGTTGGTGGAAATA-3’
	Reverse	5’-TGCTCTTCTTGGTTCCATGTC-3’
**EDEM1 (ID:9695)**	***Forward***	5’-GAATGGCTGAGGAGGAGATTAC-3’
	Reverse	5’-CTACACGTGGGAATAGGAAGATG-3’
**DERLIN1 (ID:79139)**	***Forward***	5’-GCCAATCACTGCCACCTTTT-3’
	Reverse	5’-ATAGTCTGCTGGCCTCCCAT-3’
**PER1 (ID:5187)**	***Forward***	5’-CCAGCACCACTAAGCGTAAA-3’
	Reverse	5’-TGACGGCGGATCTTTCTTG-3’
**TBP (ID:6908)**	***Forward***	5’-CTTCGGAGAGTTCTGGGATTG-3’
	Reverse	5’-AGCAAACCGCTTGGGATTA-3’
